# Delayed mortality, resistance and the sweet spot, as the good, the bad and the ugly in phosphine use

**DOI:** 10.1038/s41598-021-83463-y

**Published:** 2021-02-16

**Authors:** Evagelia Lampiri, Paraskevi Agrafioti, Christos G. Athanassiou

**Affiliations:** 1 Institute of Bio-Economy and Agri-Technology (iBO), Center for Research and Technology, 38333 Volos, Magnesia Greece; 2 Laboratory of Entomology and Agricultural Zoology, Department of Agriculture, Crop Production and Rural Environment, University of Thessaly, Phytokou str., Nea Ionia, 38446 Magnesia, Greece

**Keywords:** Plant sciences, Zoology

## Abstract

Phosphine is the most commonly used gas for fumigation for durable commodities globally, but there is still inadequate information regarding its efficacy in conjunction with proper concentration monitoring. In a series of bioassays, insect mortality after specific exposure intervals to phosphine in selected species was examined, as well as the appearance of the so called "sweet spot". The species that were tested were: *Oryzaephilus surinamensis* (L.), *Tribolium castaneum* (Herbst), *Sitophilus oryzae* (L.) and *Rhyzopertha dominica* (F.) with populations that had different levels of phosphine resistance. Evaluation was conducted by using the Phosphine Tolerance Test (PTT), with exposure of the adult stage for 15, 30, 60, 90, 150 and 300 min at 3000 ppm. At the end of these intervals (separate bioassays for each time interval), the insects were transferred to Petri dishes, in which recovery was recorded at different time intervals (2 h, 1, 2 and 7 days). The majority of susceptible populations of all species were instantly immobilized even in the shortest exposure period (15 min), in contrast with resistant populations that were active even after 300 min. After exposure to phosphine, populations and exposure time affected mortality of susceptible populations, whereas resistant populations recovered regardless of species and exposure time. Additional bioassays at the concentrations of 500, 1000, 2000 and 3000 ppm for 1, 3, 5, 20, 30 and 40 h showed the presence of the "sweet spot", i.e., decrease of mortality with the increase of concentration. In fact, for most of the tested species, the "sweet spot" appeared in 1000 and 2000 ppm at a 5-h exposure time, regardless of the level of resistance to phosphine. This observation is particularly important both in terms of the assessment of resistance and in the context of non-linear recovery at elevated concentrations, indicating the occurrence of strong hormetic reversals in phosphine efficacy.

## Introduction

Phosphine is currently the main gaseous insecticide that is applied for insect control in a wide range of durable commodities, such as cereals, legumes, oilseeds, tobacco, dried fruits and herbs, but also used in food storage and processing facilities or transport vehicles^1–5^. This gas is particularly important for global food security, as it combines efficacy against a wide range of pest species that occur at the post-harvest stages of agricultural products and food, with low application cost^6–9^. However, there are certain limitations in the use of phosphine, such as its corrosiveness to certain metals, which may irreversibly damage equipment, and its flammability at high concentrations, which may cause accidents during application^9–11^. Nevertheless, these problems can be alleviated by following best management practices in phosphine fumigation, such as proper concentration monitoring, adequate sealing of the facilities that are to be fumigated and safety measures^6,7,10–12^.

Apart from the above, the continuous and improper use of phosphine gas in many parts of the world has led to the major threat for its future use: the development of resistance^12^. Currently, there are numerous reports about the development of the so called “strong resistance” from many parts of the world^2,12–17^. Insect resistance to phosphine is a global problem that has become alarming, as resistant populations of stored product insects have been detected in most parts of the world and in at least eleven insect species^2,4,8,12,17–22^. For example, Opit et al.^19^ examined nine different populations of the red flour beetle, *Tribolium castaneum* (Herbst) (Coleoptera: Tenebrionidae) and five different populations of the lesser grain borer, *Rhyzopertha dominica* (F.) (Coleoptera: Bostrychidae), collected from different locations in Oklahoma (USA), and concluded that eight *T. castaneum* and all *R. dominica* populations were phosphine resistant. Similarly Agrafioti et al.^17^, in a survey of field insect populations from different regions of Greece, concluded that the majority of the sampled populations of the saw-toothed grain beetle, *Oryzaephilus surinamensis* (L.) (Coleoptera: Silvanidae), the rice weevil, *Sitophilus oryzae* (L.) (Coleoptera: Curculionidae), the confused flour beetle, *Tribolium confusum* Jacquelin du Val (Coleoptera: Tenebrionidae), the rusty grain beetle, *Cryptolestes ferrugineus* (Stephens) (Coleoptera: Laemophloeidae), *T. castaneum* and *R. dominica* were resistant to phosphine. Moreover, in Australia Holloway et al.^23^ diagnosed 24 populations of *S. oryzae* with strong resistance to phosphine. These reports underline the importance of this phenomenon as a global threat in stored product protection^12^.

Regarding detection and estimation of phosphine resistance, there are numerous protocols that have been developed, with often contradictory results^4,12,17,24,25^. The most commonly used protocol is that of the Food and Agriculture Organization (FAO), according to which, many of the insect species that have been tested so far, are placed in glass jars and exposed for 20 h to 30 ppm of phosphine to estimate the occurrence of resistance^19,24,26–28^. However, there are several modifications in the FAO protocol that have been proposed by different research groups, often resulting in data that are not directly comparable^8,17,21,23,29^. For example, Holloway et al.^23^ retained the exposure time proposed by the FAO protocol, varying the phosphine concentration between 0.04 mg l^−1^ and 0.25 mg l^−1^ to determine weak and strong resistance, respectively, in *S. oryzae* populations from Australia. Overall, the FAO protocol is a laborious and time-consuming method that requires specialized equipment to estimate concentrations (e.g. Gas Chromatography) and to maintain the insects (e.g. incubators), while mortality results are obtained 7–14 days after the termination of the exposure^17,19,21^. Similarly, the “dose response” bioassays are based on the exposure of insects for fixed intervals (usually 3 days) to various concentrations, ranging between 50 and 2000 ppm^4,30^. It is generally regarded that all these methods are mostly adapted for laboratory-based scientific research and have far less usefulness at the industrial scale, which requires a rapid (e.g. a same day) diagnostic tool^22^.

A recently developed rapid diagnostic method for the detection of phosphine resistance is the so called Phosphine Tolerance Test (PTT), which is a commercially available kit that has been developed by Detia Degesch GmbH (Laudenbach, Germany)^17,31^. According to this diagnostic, phosphine is generated on site, and insects are exposed for 9–16 min to 3000 ppm of phosphine. The most important difference of this diagnostic test with the dose response bioassays is that the results are based on immobilization and not on mortality, which greatly reduces the time that is needed for the final diagnosis^17,22,31^. Currently, PTT is the only commercially available rapid diagnostic test for phosphine resistance, and is a useful industry-oriented tool for fumigators.

Unlike other common insecticides where their efficacy depends on the dose (concentration) applied, in phosphine the response follows the plasticity of the relationship between exposure interval and gas concentration. This relationship mostly follows a linear pattern, which means that an increase in exposure time and phosphine concentration leads to an increase in insect mortality^12,32,33^. This linear response can be used to differentiate susceptible from resistant populations, but also to scale and quantify resistance^12^. For example, Nayak et al.^4^ was able to separate susceptible, weakly resistant and strongly resistant populations of *C. ferrugineus* sampled from Australia, based on the immobilization (knockdown) time after short exposures to phosphine. Hence, based on the above, time to immobilization can be a good indicator of resistance^4,12,17^.

The relationship between immobilization and mortality has been questioned in several papers, despite the fact that some generalization can be drawn^4,12,22^. The same holds for the term immobilization, given that some authors use “knockdown”, which is mostly related with immediate change in mobility during the exposure, as in the case of neurotoxic insecticides, such as pyrethroids^4,22,34,35^. Earlier studies used the term “narcosis” for insect immobilization after exposure to phosphine, indicating an irreversible effect that is more likely to lead to mortality than to recovery^36–38^. For phosphine, most studies are focused on immediate results, in terms of either knockdown or mortality, but there is still inadequate information for the delayed effects of phosphine exposure, i.e. the knockdown or mortality that occurs after a certain period of days or even weeks post-exposure^8,21,30,39^. For several insecticides that have been utilized for the control of stored-products, there is a considerable delayed mortality effect^40–43^. For example, Athanassiou et al.^40^ exposed adults of *R. dominica* to the bacterial insecticide spinosad for short intervals (24 h or less), and found that, despite the fact that immediate mortality was low, all adults were dead some days later in untreated wheat. Similarly, Doganay et al.^41^ exposed adults of the larger grain borer, *Prostephanus truncatus* (Horn) (Coleoptera: Bostrychidae) to pyrethroid-treated surfaces, and recorded a considerably high level of delayed mortality at short post-exposure intervals. In a recent work, Athanassiou et al.^22^ studied delayed mortality after extremely short exposures (15 and 90 min) to high concentrations of phosphine (3000 ppm) and found that susceptible individuals of *T. castaneum* were dead some days later. Nevertheless, in that study, there was a noticeable recovery of resistant *T. castaneum* adults after exposure, despite their initial immobilization^22^. Hence, it becomes uncertain if immediate behavioral changes after exposure to phosphine may be utilized for prediction of delayed mortality, and, as a result, the concomitant susceptibility level of a given population^12^.

Interestingly, it has been often observed that the relationship of exposure time and phosphine concentration is linear only up to a certain threshold, beyond which a further increase in exposure time or a further increase in phosphine concentration results in a decrease in the percentage of immobilized insects^36,37^. This paradoxical phenomenon is called the "sweet spot" of phosphine, which is quite unusual in the case of other insecticides^12,22,34,36,37^. In fact, this peculiar phenomenon can be manifested independently of the level of population resistance to phosphine, and may appear much more often than initially reported. For example, Winks et al.^36^ and Winks & Waterford^38^ found that this phenomenon was observed in both resistant and susceptible *T. castaneum* populations when exposed to a wide range of high phosphine concentrations. Moreover, in that studies, the authors found that at elevated phosphine concentrations the time required to produce 100% mortality was longer than that for lower concentrations, indicating that the “sweet spot” may be related to reduced susceptibility^36,38^. From a practical point of view, the non-linearity of concentration and exposure may meet with several implications in “real world” applications, as elevated concentrations may lead to increased survival, rather than increased mortality^12,44,45^. The causals of this phenomenon are poorly understood, particularly under the prism of the occurrence of resistance.

Based on the above, and taking into account the data gaps on the contribution of the above phenomena to resistance development, as well as the factors that contribute to their expression, we have carried out laboratory bioassays by using a wide range of stored-product insect populations with different levels of susceptibility to phosphine. In this context, we examined if immobilization can be used as an indicator of resistance, using different exposure intervals, concentrations and post-exposure periods. At the same time, we have evaluated the factors that affect the occurrence of the “sweet spot” and its interaction with insect susceptibility to phosphine.

## Results

### Relationship between narcosis and recovery

**Figure 1:**
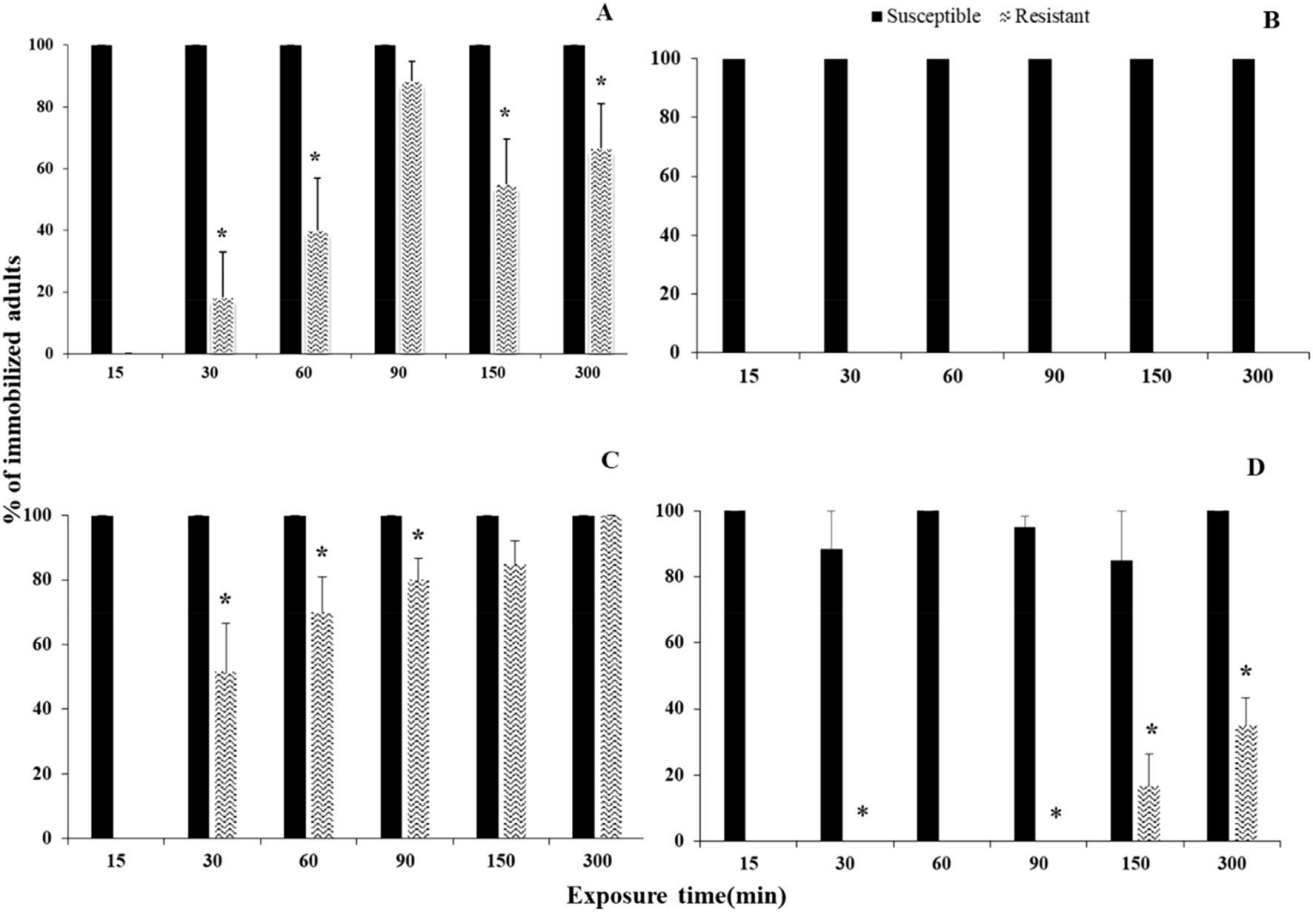
Percentage (% ± SE) of immobilized adults of two populations (phosphine-susceptible and -resistant) of *R. dominica* (**A**), *S. oryzae* (**B**), *O. surinamensis* (**C**) and *T. castaneum* (**D**), after different exposure times to phosphine at 3000 ppm (in min). Means with asterisks indicated differences between susceptible and resistant populations according to Student’s *t*-test, at n-2 degrees of freedom and at 0.05. According to *t*-test, the parameters for each exposure interval were: for *R. dominica* at 30 min t = 5.5, *P* < 0.01, at 60 min t = 3.5, *P* < 0.01, at 150 min t = 3.0, *P* < 0.01, at 300 min t = 2.2, *P* < 0.01, for *O. surinamensis* at 30 min t = 3.2, *P* < 0.01, at 60 min t = 2.7, *P* < 0.01, at 90 min t = 2.9, *P* < 0.01, for *T. castaneum* at 30 min t = 7.5, *P* < 0.01, at 90 min t = 27.8, *P* < 0.01, at 150 min t = 3.8, *P* < 0.01, at 300 min t = 7.6, *P* < 0.01. In all cases, *df* value was 10.

For all species, all main effects and interactions were significant (Table 1). The insect populations examined in our experiment did not respond in the same way to the different phosphine exposure times (Fig. 1). Comparing the susceptible and resistant population of each species at different exposure times, we recorded significant differences in most of the cases tested. For the susceptible populations, all adults were completely immobilized after the 15-min exposure interval. Nevertheless, for *T. castaneum*, there were some deviations in immobilization at longer exposures, due to a small number of adults that indicated some temporary movement (Fig. 1D). In contrast, immobilization was low for the resistant populations; in fact, there was no immobilization of the resistant *S. oryzae* population, for the entire observation period (Fig. 1B). Interestingly, for the resistant *R. dominica* population, although the percentage of immobilization was gradually increased with the increase of the exposure period, we had more adults immobilized at 90 min, as compared with 150 min (Fig. 1A).

**Table 1:** Generalized linear model at 3000 ppm of phosphine, showing the effects of the exposure time and the post exposure time, for each population and species.

Effect	*Rhyzopertha dominica*			*Sitophilus oryzae*			*Oryzaephilus surinmaneis*			*Tribolium castaneum*		
	df	Wald x^2^	*P*	df	Wald x^2^	*P*	df	Wald x^2^	*P*	df	Wald x^2^	*P*
Population	1	2846.7	< 0.001	1	797.0	< 0.001	1	1565.9	< 0.001	1	2246.1	< 0.001
Exposure time	5	114.9	< 0.001	5	34.9	< 0.001	5	2212.9	< 0.001	5	926.1	< 0.001
Post exposure time	3	843.7	< 0.001	3	611.5	< 0.001	3	556.7	< 0.001	3	181.6	< 0.001
Population × exposure time	5	135.8	< 0.001	3	18.1	< 0.001	5	969.5	< 0.001	4	93.9	< 0.001
Exposure time × post exposure time	15	152.0	< 0.001	15	269.9	< 0.001	15	437.3	< 0.001	15	268.0	< 0.001
Population × exposure time × post exposure time	16	1000.4	< 0.001	–*	–	–	16	378.7	< 0.001	12	555.1	< 0.001

*Interactions could not be estimated.

**Figure 2:**
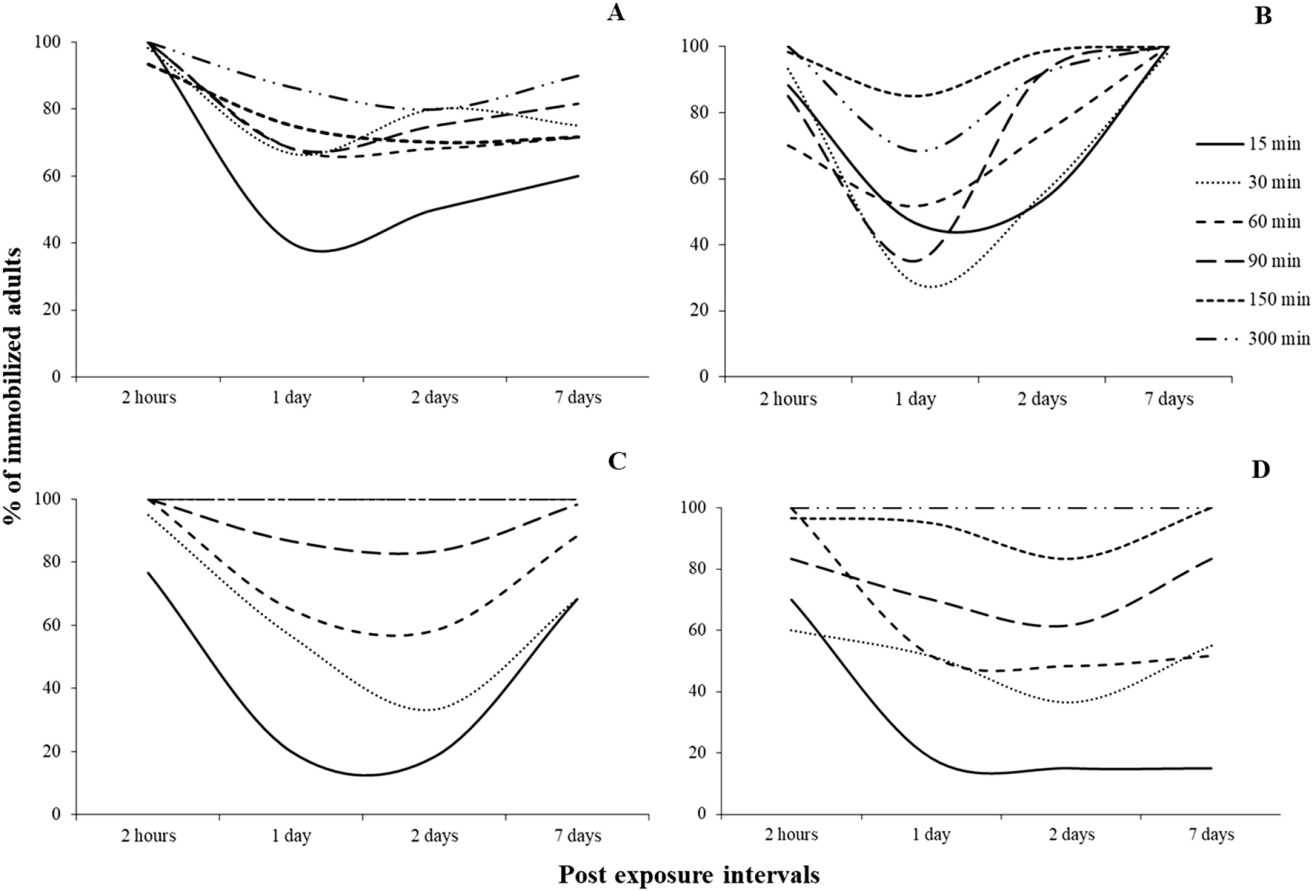
Percentage (%) of immobilized adults of laboratory populations of *R. dominica* (**A**), *S. oryzae* (**B**), *O. surinamensis* (**C**) and *T. castaneum* (**D**), for each exposure interval (15, 30, 60, 90, 150 and 300 min) after exposure to phosphine at 3000 ppm for different post exposure intervals (2 h, 1, 2 and 7 days). The equations for each exposure interval for each species are presented in Table 3.

**Figure 3:**
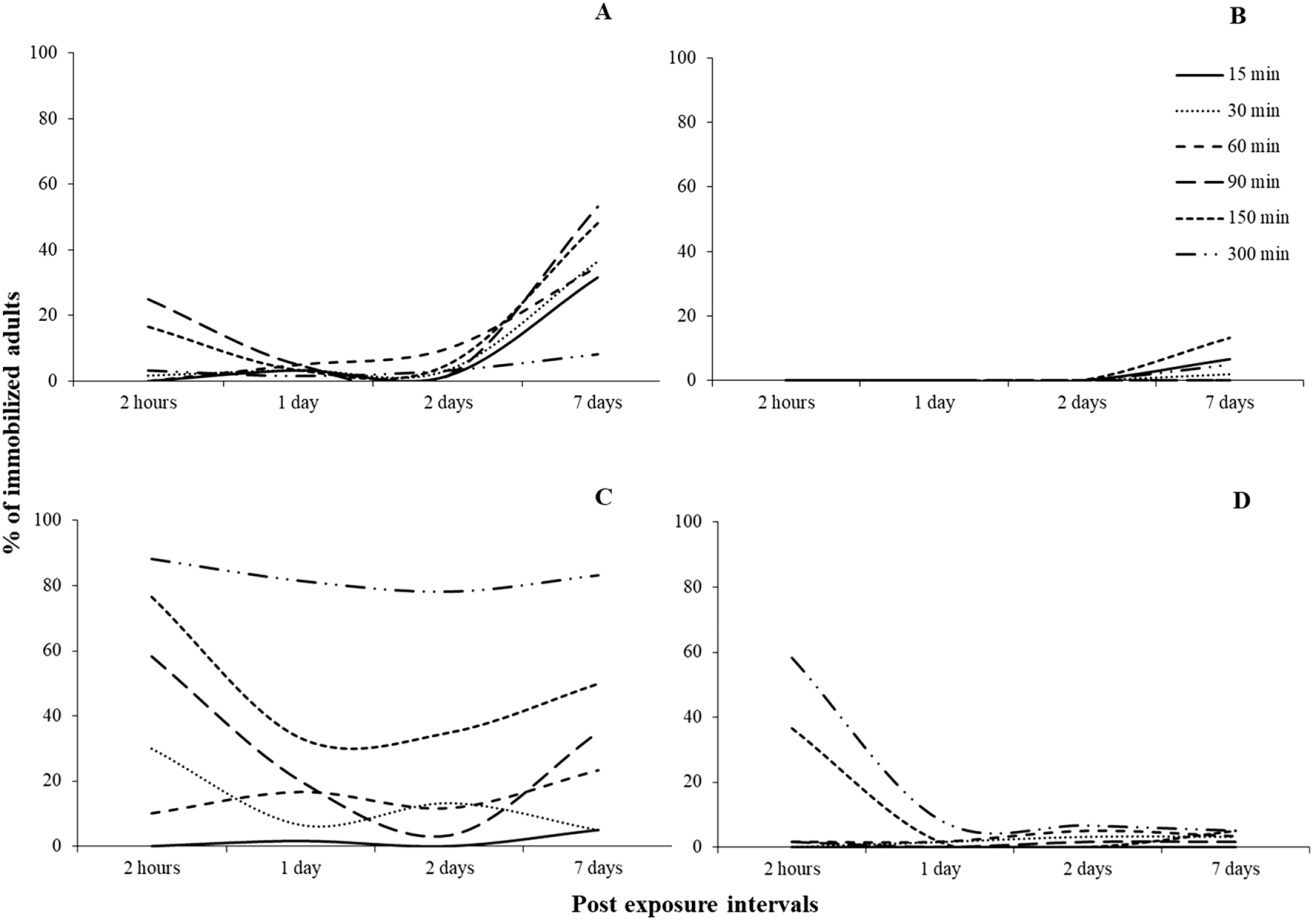
Percentage (%) of immobilized adults of resistant populations of *R. dominica* GA6 (**A**), *S. oryzae* 3TAB (**B**), *O. surinamensis* ASC11 (**C**) and *T. castaneum* BTS (**D**) for each exposure interval (15, 30, 60, 90, 150 and 300 min) after exposure to phosphine at 3000 ppm for different post exposure intervals (2 h, 1, 2 and 7 days). The equations for each exposure interval for each species are presented in Table 3.

The non-linearity in adult immobilization response was more evident at the post-exposure periods for both susceptible and resistant populations (Figs. 2 and 3). In the majority of the cases, the exposure-immobilization relationship was better described by either cubic or quadratic regressions (Table 2). Regarding the susceptible populations, we observed a temporary decrease of the percentage of immobilized adults one day after the termination of the exposure, but only in some of the combinations tested (Fig. 2). This phenomenon was expressed much more vigorously in adults that had been exposed for short intervals, i.e. 15–60 min, while, for longer exposure intervals, such as 300 min, the response tends to become more linear. Moreover, for some short exposures, this reduction occurs later, i.e. 2 days after the termination of the exposure, such as in the susceptible *O. surinamensis* population for the 30 min interval (Fig. 2C), or does not occur at all, such as in the susceptible *T. castaneum* for the 15 min interval (Fig. 2D).

**Table 2:** Results of immediate effects, for curve estimations for each population tested, exposed on different exposure times (15, 30, 60, 90, 150 and 300 min) at 3000 ppm of phosphine.

Populations	Exposure time (min)	Equation	F	*P* value	df	R
*R. dominica* Lab	15	Cubic	8.1	< 0.01	3.23	0.74
	30	Cubic	5.1	< 0.01	3.23	0.66
	60	Cubic	22.6	< 0.01	3.23	0.88
	90	Cubic	7.1	< 0.01	3.23	0.88
	150	Quadratic	2.8	0.08	2.23	0.46
	300	Quadratic	4.7	< 0.01	2.23	0.55
*S. oryzae* Lab	15	Cubic	7.3	< 0.01	3.23	0.72
	30	Cubic	27.2	< 0.01	3.23	0.89
	60	Cubic	6.2	< 0.01	3.23	0.69
	90	Cubic	57.6	< 0.01	3.23	0.94
	150	Cubic	6.3	< 0.01	3.23	0.70
	300	Cubic	15.2	< 0.01	3.23	0.84
*O. surinamensis* Lab	15	Cubic	15.3	< 0.01	3.23	0.83
	30	Quadratic	20.1	< 0.01	2.23	0.81
	60	Quadratic	16.6	< 0.01	2.23	0.78
	90	Cubic	3.8	0.02	3.23	0.60
	150	–*	–	–	–	–
	300	–	–	–	–	–
*T. castaneum* Lab	15	Cubic	1.5	0.23	3.23	0.43
	30	Cubic	2.1	0.13	3.23	0.49
	60	Linear	2.4	0.13	1.23	0.31
	90	Quadratic	33.8	< 0.01	2.23	0.87
	150	Cubic	9.2	< 0.01	3.23	0.76
	300	Quadratic	1.3	0.28	2.23	0.33
*R. dominica* GA6	15	Quadratic	15.4	< 0.01	2.23	0.77
	30	Quadratic	16.4	< 0.01	2.23	0.78
	60	Linear	136.5	< 0.01	1.23	0.92
	90	Quadratic	30.4	< 0.01	2.23	0.86
	150	Quadratic	8.3	< 0.01	2.23	0.66
	300	Quadratic	2.3	0.12	2.23	0.42
*S. oryzae* 3TAB	15	Quadratic	6.2	< 0.01	2.23	0.61
	30	Quadratic	3.9	0.03	2.23	0.52
	60	–	–	–	–	–
	90	–	–	–	–	–
	150	Quadratic	2.4	0.10	2.23	0.43
	300	–	–	–	–	–
*O. surinamensis* ASC11	15	Cubic	1.5	0.23	3.23	0.43
	30	Cubic	2.1	0.13	3.23	0.49
	60	Linear	2.4	0.13	1.23	0.31
	90	Quadratic	33.8	< 0.01	2.23	0.87
	150	Cubic	9.2	< 0.01	3.23	0.76
	300	Quadratic	1.3	0.28	2.23	0.33
*T. castaneum* BTS	15	–	–	–	–	–
	30	Cubic	0.87	0.47	3.23	0.34
	60	Cubic	0.67	0.57	3.23	0.30
	90	Cubic	0.33	0.80	3.23	0.21
	150	Cubic	10.1	< 0.01	3.23	0.77
	300	Cubic	32.7	< 0.01	3.23	0.91

*Could not be estimated accurately.

This same trend is expressed for the resistant populations, but following a different pattern (Fig. 3). In general, at the post-exposure intervals, the percentages of the immobilized adults were lower than the respective figures of the susceptible populations, but had the same non-linear response, that differed among species. Hence, for *T. castaneum*, a considerable percentage of the exposed adults was found to be immobilized at the two highest exposure intervals right after the termination of the exposure, while for the rest of the exposures immobilization was negligible (Fig. 3D). Nevertheless, the increased immobilization at these two long exposures was temporal, and dropped to values close to zero 1 d after the exposure. In contrast, for *S. oryzae*, there was no immobilization right after the termination of the exposure, regardless of the exposure interval, but there was eventually a small percentage of adults classified as immobilized seven days later (Fig. 3B). The resistant *R. dominica* and *O. surinamensis* populations indicated non-linear immobilization response, with a reduction that was observed, either on the first or the second day after the exposure, depending on the species-exposure-interval-post exposure period. For *R. dominica*, and in most post-exposure periods, there was a temporary decrease in the percentage of immobilized adults at the 1 and 2-days intervals, followed by a subsequent increase at the end of the observation period (Fig. 3A). Similar trends were noted for *O. surinamensis*, where percentages of immobilization were much higher, while this non-linear response was proportional to the increase of the post-exposure period (Fig. 3C).

### Occurrence of the “sweet spot”

**Figure 4:**
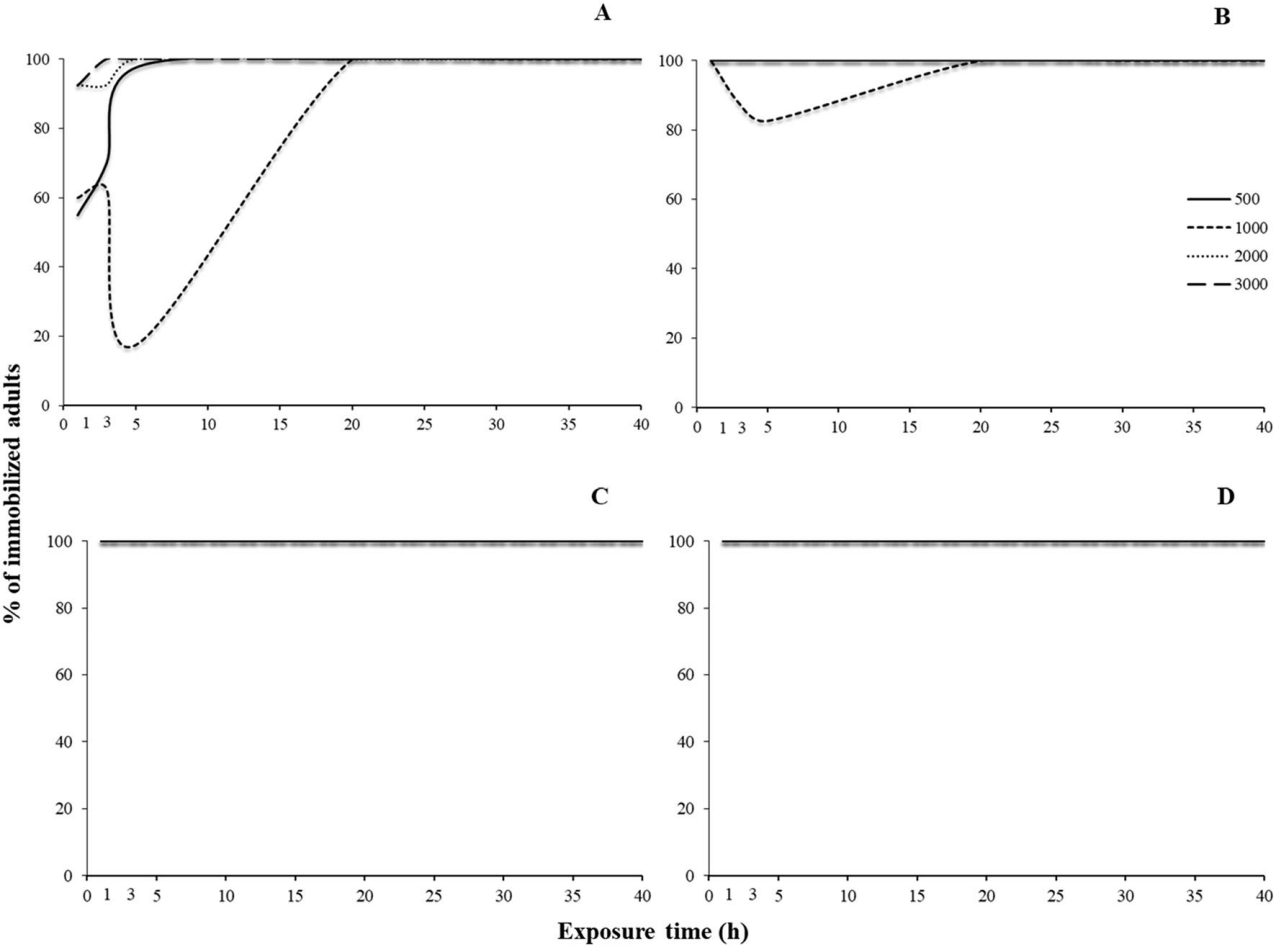
Percentage (%) of immobilized adults of laboratory populations of *R. dominica* (**A**), *S. oryzae* (**B**), *O. surinamensis* (**C**) and *T. castaneum* (**D**), for different exposure time (1, 3, 5, 20, 30 and 40 h) and different concentrations of phosphine (500, 1000, 2000 and 3000 ppm). The equations for each exposure interval for each species are presented in Table 6.

For most of the species tested, all main effects and interactions were significant, with few exceptions in *S. oryzae* (Tables 3 and 4). Regarding the susceptible populations, right after the termination of the exposure, immobilization varied among species. For *R. dominica*, the increase of exposure increased immobilization for all concentrations tested, with one sole exception: the exposure at 1000 ppm for 5 h (Fig. 4A). At 1000 ppm, after 3 h of exposure, immobilization was 60%, while 2 h later this percentage dropped to 20%, and was back up to 100% at the 20 h. Similarly, the same phenomenon was recorded at exactly the same exposure and concentration combination for *S. oryzae*, while immobilization was 100% in all other combinations (Fig. 4B). In contrast, for *O. surinamensis* and *T. castaneum*, all adults were immobilized, regardless of the exposure and concentration (Fig. 4C,D).

**Table 3:** Generalized linear model showing the effects of different exposure times and concentrations (500, 1000, 2000 and 3000 ppm) for immediate effects, for each species and population.

Effect	*Rhyzopertha dominica*			*Sitophilus oryzae*			*Oryzaephilus surinmaneis*			*Tribolium castaneum*		
	df	Wald x^2^	*P*	df	Wald x^2^	*P*	df	Wald x^2^	*P*	df	Wald x^2^	*P*
Population	1	825.4	< 0.001	1	450.9	< 0.001	1	82.1	< 0.001	1	186.8	< 0.001
Exposure time	5	1595.6	< 0.001	5	505.9	< 0.001	5	120.0	< 0.001	5	347.2	< 0.001
Concentration	3	274.5	< 0.001	3	9.2	0.027	3	25.1	< 0.001	3	148.8	< 0.001
Population x Exposure time	5	897.3	< 0.001	4	474.7	< 0.001	5	120.0	< 0.001	5	347.3	< 0.001
Population x Concentration	3	68.4	< 0.001	3	3.9	0.322	3	25.1	< 0.001	3	146.0	< 0.001
Exposure time x Concentration	15	364.3	< 0.001	15	105.3	< 0.001	15	66.1	< 0.001	15	266.6	< 0.001
Population x Exposure time x concentration	12	113.4	< 0.001	8	95.1	< 0.001	15	66.9	< 0.001	11	266.6	< 0.001

**Table 4:** Generalized linear model showing the effects of different exposure times and concentrations (500, 1000, 2000 and 3000 ppm) for post exposures, for each species and population tested.

Effect	*Rhyzopertha dominica*			*Sitophilus oryzae*			*Oryzaephilus surinmaneis*			*Tribolium castaneum*		
	df	Wald x^2^	*P*	df	Wald x^2^	*P*	df	Wald x^2^	*P*	df	Wald x^2^	*P*
Population	1	955.6	< 0.001	1	1075.0	< 0.001	1	368.3	< 0.001	1	749.1	< 0.001
Exposure time	5	2419.0	< 0.001	5	2196.2	< 0.001	5	913.3	< 0.001	5	1123.8	< 0.001
Concentration	3	6.1	< 0.001	3	44.5	0.027	3	16.7	< 0.001	3	172.0	< 0.001
Population x Exposure time	5	855.1	< 0.001	5	1130.3	< 0.001	5	448.7	< 0.001	5	817.3	< 0.001
Population x Concentration	3	100.4	< 0.001	3	83.6	0.322	3	10.1	0.017	3	131.3	< 0.001
Exposure time x Concentration	15	102.8	< 0.001	15	84.1	< 0.001	15	68.4	< 0.001	15	355.5	< 0.001
Population x Exposure time x Concentration	15	160.9	< 0.001	15	316.7	< 0.001	15	60.0	< 0.001	11	280.9	< 0.001

**Figure 5:**
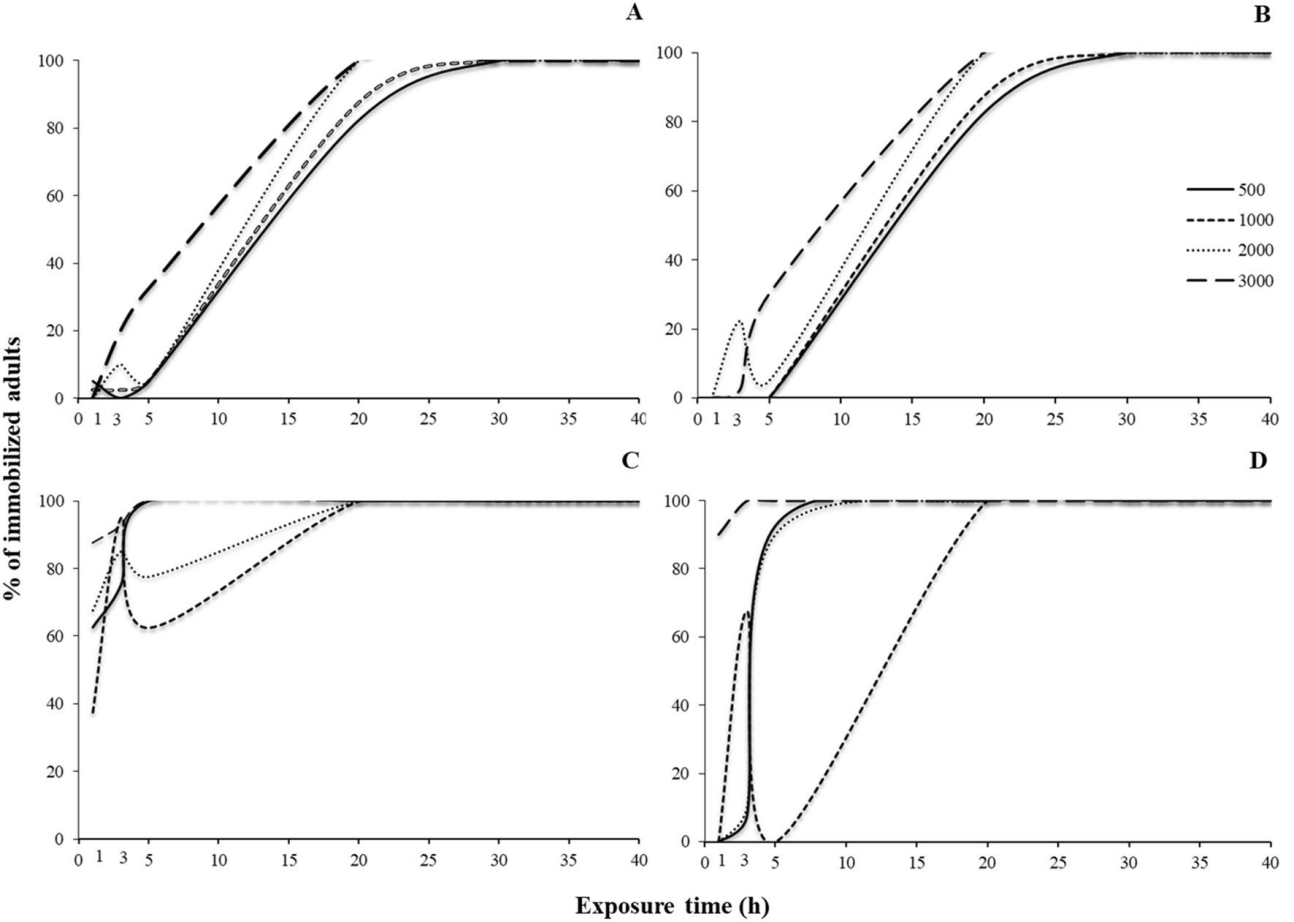
Percentage (%) of immobilized adults of resistant populations of *R. dominica* GA6 (**A**), *S. oryzae* 3TAB (**B**), *O. surinamensis* ASC11 (**C**) and *T. castaneum* BTS (**D**), for different exposure time (1, 3, 5, 20, 30 and 40 h) and different concentrations of phosphine (500, 1000, 2000 and 3000 ppm). The equations for each exposure interval for each species are presented in Table 6.

**Figure 6:**
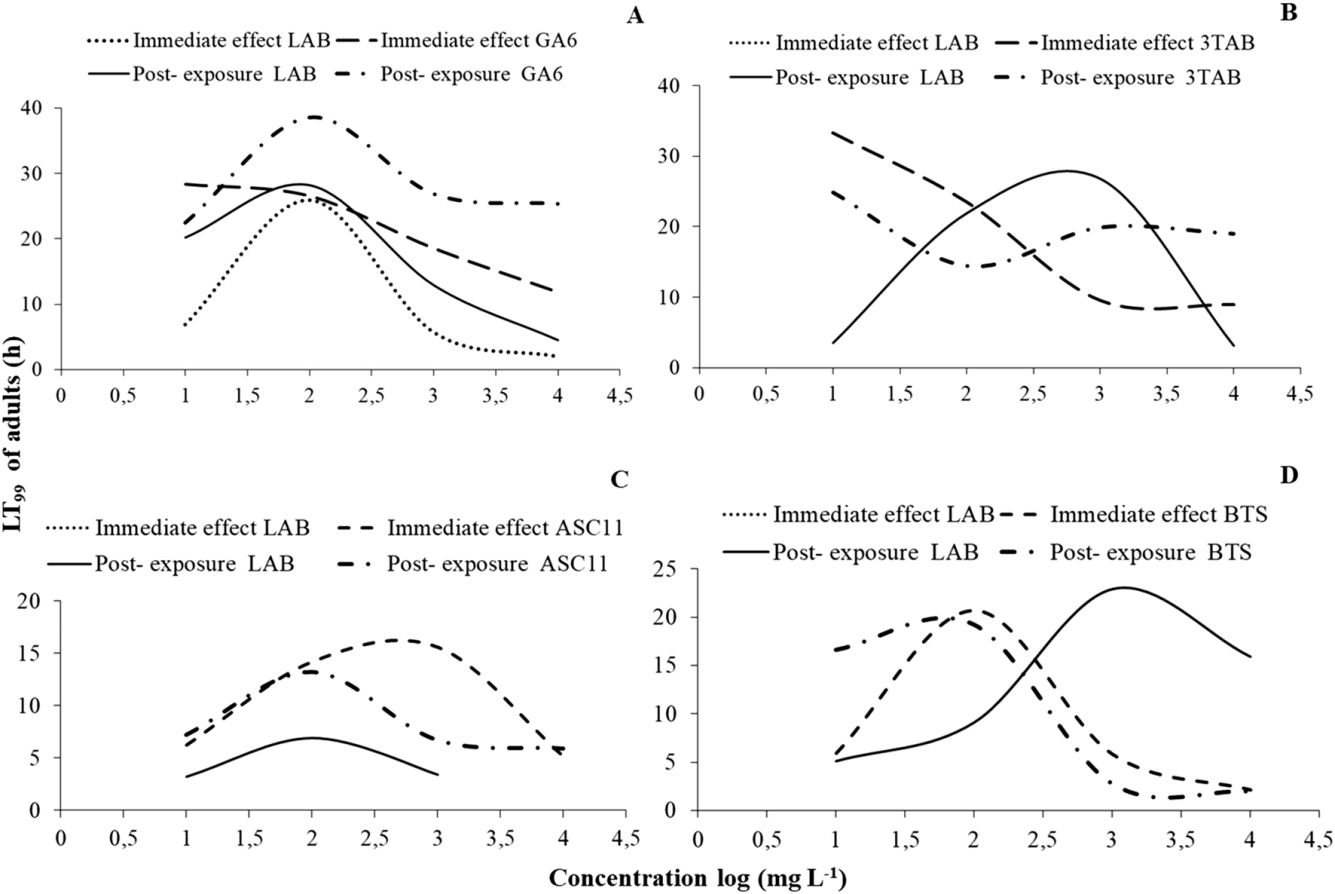
LT_99_ of adults of two populations (phosphine-susceptible and –resistant) of *R. dominica* (**A**), *S. oryzae* (**B**), *O. surinamensis* (**C**), *T. castaneum* (**D**), after exposure to different concentrations of phosphine (immediate effect) and after 7 days (post exposure). Were no lines exist, LT_99_ could not be estimated.

The sweet spot was also recorded in the case of the resistant populations (Fig. 5). For *R. dominica* and *S. oryzae*, as above, a temporary decrease in the percentage of the immobilized adults was recorded when insects were exposed for 5 h at 2000 ppm, and then increased again at longer exposures (Fig. 5A,B). For *O. surinamensis*, however, the sweet spot was recorded at the same interval (5 h), but at two concentrations, 1000 and, to a lesser extent, 2000 ppm, while in all other combinations the increase of the exposure and the concentration resulted in increased immobilization (Fig. 5C). Interestingly, for *T. castaneum*, this phenomenon was impressively expressed at 1000 ppm after 5 h of exposure, as there was no adult immobilization, while 2 h earlier, immobilization was 60% (7 immobilized adults), reaching 100% again at the 20 h exposure (Fig. 5D). Considering the LT_99_ of the exposed adults, we saw that, with the exception of *R. dominica*, this could be estimated only for the resistant populations (Fig. 6). Moreover, the changes of the values of LT_99_ were not linear with the increase of phosphine concentration, but, at least for the resistant populations of *O. surinamensis* and *T. castaneum*, and the susceptible *R. dominica* population, increased with the increase of log concentration, up to a certain point (Fig. 6A,C,D). In most of the cases, this relationship was better described by significant cubic, quadratic and inverse regressions (Table 5).

**Table 5:** Sweet spot curve estimations for immediate effects, of insects exposed on different concentrations (500, 1000, 2000 and 3000 ppm) for all exposure intervals (1, 3, 5, 20, 30 and 40 h), for each species and population.

Populations	Concentration (ppm)	Equation	F	*P* value	df	R
*R. dominica Lab*	500	Inverse	42.6	< 0.01	1.23	0.88
	1000	Quadratic	12.0	< 0.01	2.23	0.80
	2000	Inverse	124.1	< 0.01	1.23	0.92
	3000	Inverse	12.5	< 0.01	1.23	0.70
*S. oryzae Lab*	500	–*	–	–	–	–
	1000	Cubic	3.0	0.05	3.23	0.56
	2000	–	–	–	–	–
	3000	–	–	–	–	–
*O. surinamensis Lab*	500	–	–	–	–	–
	1000	–	–	–	–	–
	2000	–	–	–	–	–
	3000	–	–	–	–	–
*T. castaneum Lab*	500	–	–	–	–	–
	1000	–	–	–	–	–
	2000	–	–	–	–	–
	3000	–	–	–	–	–
*R. dominica GA6*	500	Quadratic	221.7	< 0.01	2.23	0.98
	1000	Quadratic	256.6	< 0.01	2.23	0.98
	2000	Quadratic	223.9	< 0.01	2.23	0.98
	3000	Quadratic	102.0	< 0.01	2.23	0.96
*S. oryzae 3TAB*	500	Quadratic	210.8	< 0.01	2.23	0.98
	1000	Quadratic	261.9	< 0.01	2.23	0.98
	2000	Cubic	184.8	< 0.05	3.23	0.98
	3000	Quadratic	184.8	< 0.01	2.23	0.98
*O. surinamensis ASC11*	500	Inverse	18.3	< 0.01	1.23	0.67
	1000	Inverse	45.8	< 0.01	1.23	0.88
	2000	Logarithmic	110.9	< 0.01	1.23	0.94
	3000	Logarithmic	8.9	< 0.01	1.23	0.87
*T. castaneum BTS*	500	Cubic	29.1	< 0.01	3.23	0.98
	1000	Quadratic	23.8	< 0.01	2.23	0.98
	2000	Cubic	33.3	< 0.01	3.23	0.98
	3000	Inverse	28.0	< 0.01	1.23	0.98

*Equations could not be estimated.

**Figure 7:**
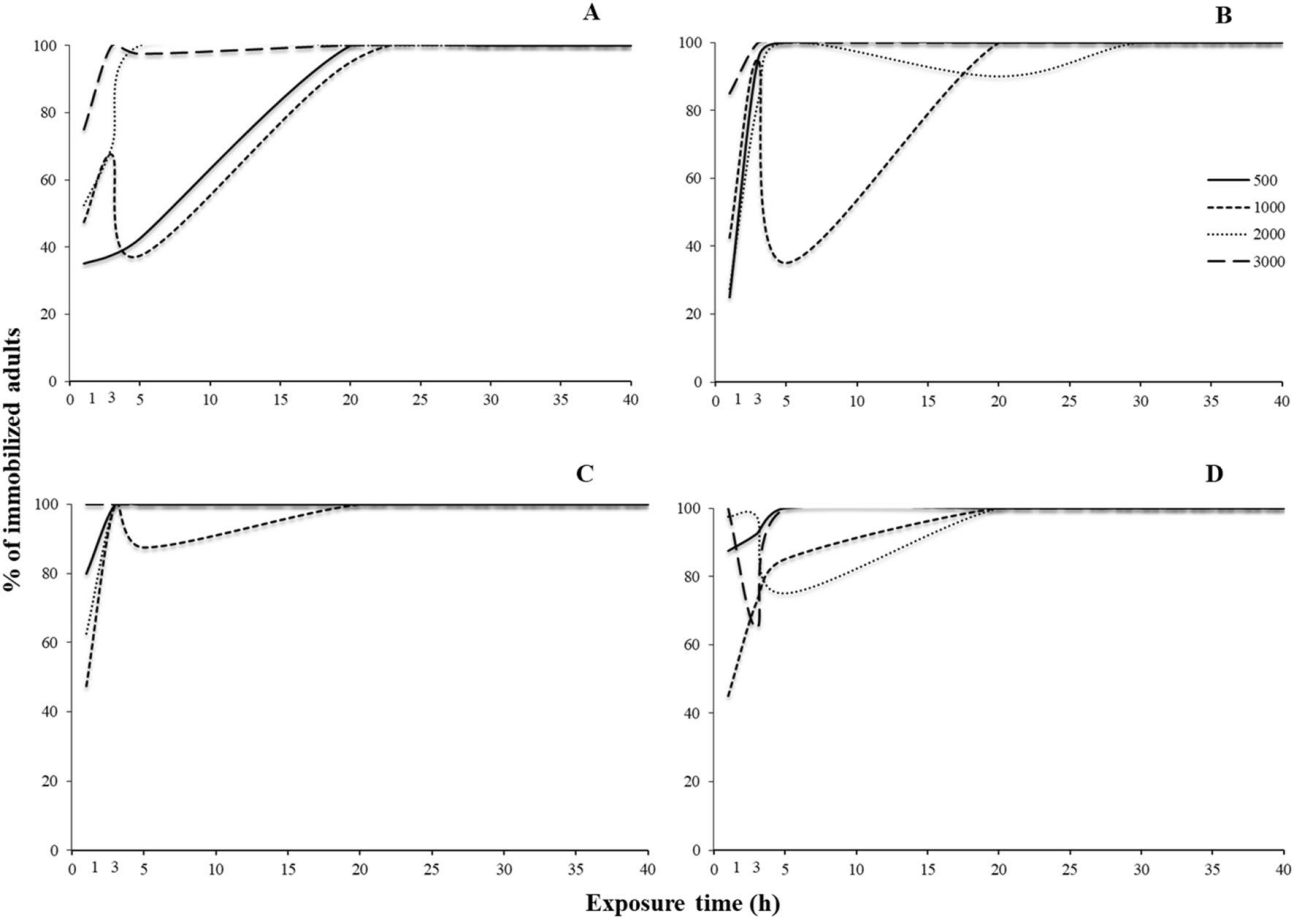
Percentage (%) of immobilized adults of laboratory populations of stored product beetle species, for different exposure times (1, 3, 5, 20, 30 and 40 h) and different concentrations of phosphine, i.e. 500 (**A**), 1000 (**B**), 2000 (**C**) and 3000 ppm (**D**) after 7 days. The equations for each exposure interval and each species are presented in Table 7.

For 500 and 3000 ppm, delayed immobilization was increased with the increase of the exposure interval, for all susceptible populations (Fig. 7). In contrast, at 1000 ppm, there was a noticeable decrease in immobilization for *R. dominica* adults that had been exposed for 5 h, as compared with shorter or longer exposures (Fig. 7A). Similar trends were noted for *S. oryzae* at 1000 ppm, but to a far less extent (Fig. 7B). Moreover, smaller sweet spots were also observed at short exposure intervals at 2000 ppm (Fig. 7).

**Figure 8:**
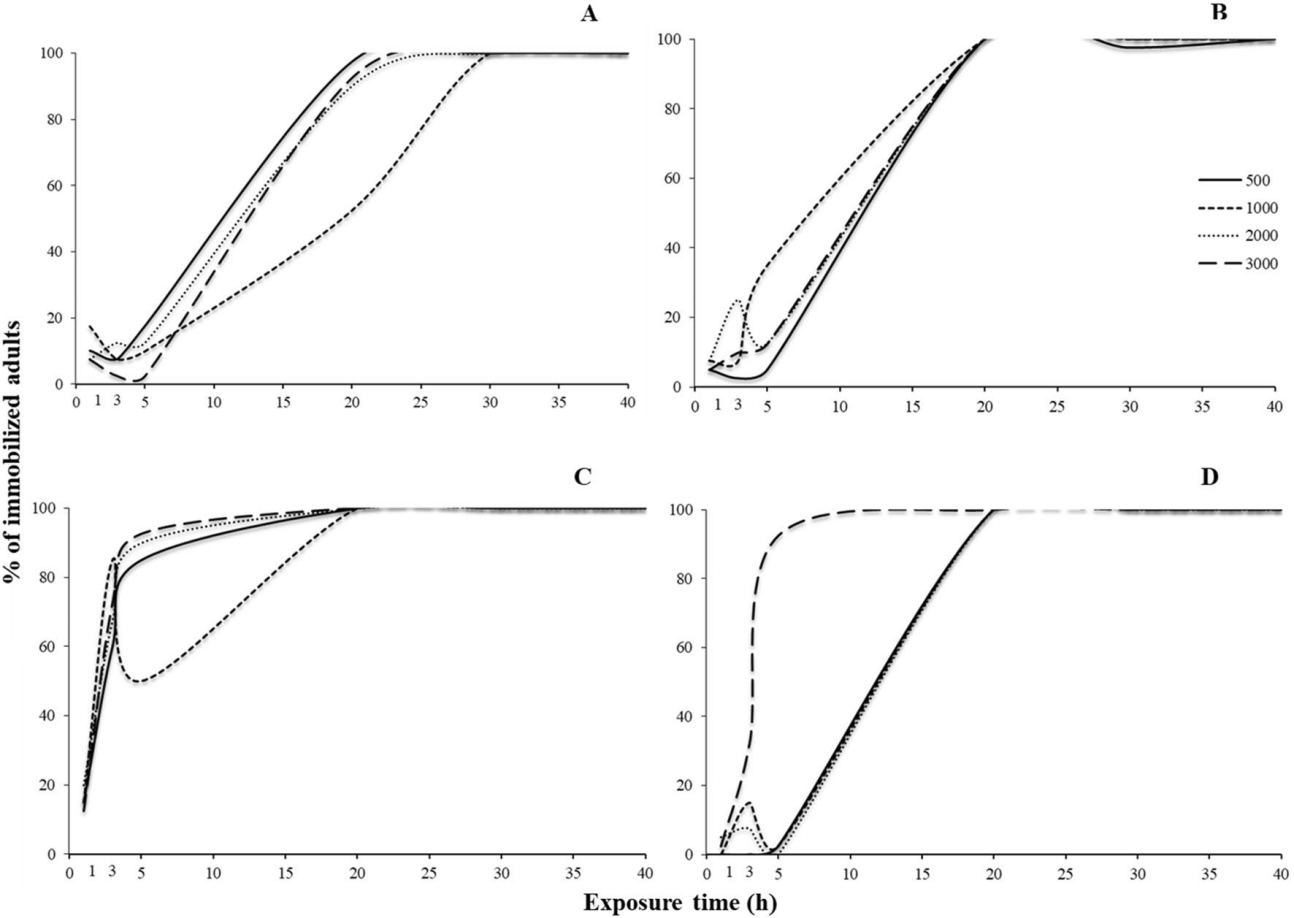
Percentage (%) of immobilized adults of resistant populations of stored product beetle species, for different exposure times (1, 3, 5, 20, 30 and 40 h) and different concentrations of phosphine, i.e. 500 (**A**), 1000 (**B**), 2000 (**C**) and 3000 ppm (**D**) after 7 days. The equations for each exposure interval for each species are presented in Table 7.

As noted for the susceptible populations, the immobilization results for the resistant populations were rather similar for 500 and 3000 ppm (Fig. 8). Conversely, at 1000 ppm, for *O. surinamensis*, immobilization was reduced at 5 h, as compared with 3 h, and also with 20 h or longer (Fig. 8C). As above, this relationship was better described by significant cubic, quadratic and inverse regressions, with some exceptions, where logarithmic and S regression provided a greater goodness of fit (Table 6).

**Table 6:** Sweet spot curve estimations for delayed effects, of insects exposed on different concentrations (500, 1000, 2000 and 3000 ppm) throughout the different exposure intervals, for each species and population.

Populations	Concentration (ppm)	Equation	F	*P* value	df	R
*R. dominica Lab*	500	Cubic	19.7	< 0.01	3.23	0.88
	1000	Quadratic	26.4	< 0.01	2.23	0.88
	2000	Logarithmic	80.2	< 0.01	1.23	0.89
	3000	Inverse	52.6	< 0.01	1.23	0.88
*S. oryzae Lab*	500	S	94.0	< 0.01	1.23	0.90
	1000	Logarithmic	18.8	< 0.01	1.23	0.67
	2000	Inverse	191.2	< 0.01	1.23	0.96
	3000	Inverse	83.3	< 0.01	1.23	0.88
*O. surinamensis Lab*	500	Inverse	35.6	< 0.01	1.23	0.88
	1000	Inverse	80.7	< 0.01	1.23	0.89
	2000	Inverse	33.5	< 0.01	1.23	0.89
	3000	–*	–	–	–	–
*T. castaneum Lab*	500	Inverse	18.2	< 0.01	1.23	0.67
	1000	Inverse	89.7	< 0.01	1.23	0.89
	2000	–	–	–	–	–
	3000	–	–	–	–	–
*R. dominica GA6*	500	Cubic	119.1	< 0.01	3.23	0.98
	1000	Cubic	76.1	< 0.01	3.23	0.96
	2000	Quadratic	222.6	< 0.01	2.23	0.98
	3000	Quadratic	195.8	< 0.01	2.23	0.98
*S. oryzae 3TAB*	500	Quadratic	189.0	< 0.01	2.23	0.98
	1000	Quadratic	225.9	< 0.01	2.23	0.98
	2000	Quadratic	153.6	< 0.01	2.23	0.98
	3000	Quadratic	178.2	< 0.01	2..23	0.98
*O. surinamensis ASC11*	500	Inverse	243.7	< 0.01	1.23	0.97
	1000	Inverse	66.6	< 0.01	1.23	0.86
	2000	Inverse	279.9	< 0.01	1.23	0.97
	3000	Inverse	426.4	< 0.01	1.23	0.98
*T. castaneum BTS*	500	Quadratic	280.1	< 0.01	2.23	0.98
	1000	Quadratic	187.6	< 0.01	2.23	0.98
	2000	Quadratic	183.5	< 0.01	2.23	0.98
	3000	Inverse	123.0	< 0.01	1.23	0.93

*Equations could not be estimated.

## Discussion

Τhe two phenomena used here are different in their frequency of expression. While the delayed effects and the immobilization- recovery patterns can be used to differentiate susceptible from resistant populations, in terms of different expressions in resistant populations, the sweet spot is rather a “global” phenomenon, as it is expressed almost equally vigorously regardless of the resistance level of the specific population that is examined. This is particularly important in the case of the use of phosphine in general, as short exposures trigger “umbrella/inverse umbrella peaks”, which can be attributed to self-regulated process^46–48^. In a recent study, Franco-Pereira et al.^48^ have modelled the “umbrella peak” phenomenon of adults of a single phosphine resistant *T. castaneum* population, under the context of binary dose–response variables. In fact, this binary, i.e. biphasic relationship, characterized by a reversal in response between low and high doses of insecticides, has been often described as “hormesis”^46,47,49^. This phenomenon is often, but not always necessarily, manifested at low concentrations of insecticides, that lie below the so called “No Observable Effects Concentration” (NOEC)^49^. However, in our study we saw serious chances in immobilization and eventual mortality patterns at elevated concentrations, which were applied at relatively short intervals, which is sufficiently different than the low dose simulation and high dose inhibition approach of the typical hormetic responses. The contribution of this phenomenon to resistance development is now broadly recognized, as it is directly linked to specific adaptation mechanisms that lead to pest resurgence^47,49^. This phenomenon is widely studied in the case of many different types of neurotoxic insecticides, but there is still inadequate information regarding non-neurotoxic compounds^46^. For instance, Guedes et al.^50^ found strong hormetic adaptations in a population of the maize weevil, *Sitophilus zeamais* Motschulsky (Coleoptera: Curculionidae) that was resistant to the pyrethroid deltamethrin. In general, this phenomenon is currently recognized as a general toxicological phenomenon, rather than a fitness cost response that is related with resistance^49^. To our knowledge, this is the first time that this biphasic relationship is studied for a wide range of species and populations for the fumigant phosphine. Guedes et al.^47^ hypothesized that insecticides with a mode of action that involves oxidative stress, such as phosphine, are ideal candidates to further investigate this phenomenon, due to the involvement of energy balance disorders that may reveal early hormetic adaptation patterns. The present data support this hypothesis.

Interestingly, the sweet spot is expressed in the same way in both intervals examined, i.e. right after the exposure and at the 7 d post-exposure interval, suggesting that short exposures to elevated phosphine concentrations cause a considerable delayed effect. At the same time, our results indicate that initial immobilization is correlated with delayed effects. For a phosphine-susceptible *T. castaneum* population, Athanassiou et al.^11^ found that exposures as short as 15–90 min caused almost complete (100%) delayed mortality to the exposed adults 7 days later. In contrast, delayed mortality of a resistant *T. castaneum* population was negligible^11^. On the other hand, Nayak et al.^4^ proposed a rapid test that is based on short exposures that last approx. 5 h, which can be used as a diagnostic to separate strongly from weakly resistant populations of *C. ferrugineus,* according to a 14 days evaluation period. In a recent study, Gourgouta et al.^51^ exposing individuals of the khapra beetle, *Trogoderma granarium* Everts (Coleoptera: Dermestidae) indicated similar post exposure results for either 7 or 14 days. Based on the “quick immobilization equals to increased susceptibility” approach, Athanassiou et al.^11^ developed a rapid diagnostic that lasts up to 15 min, which can be used to distinguish resistant from susceptible populations for thirteen different stored product beetle species. The results of the present study clearly display a considerable level of delayed mortality that is not usually evaluated in efficacy trials. Hence, adult beetles that are still active after fumigations in “real world” applications may falsely lead to the conclusion of the occurrence of resistance, while in reality, these beetles may exhibit an increased delayed mortality several days after the termination of the exposure. These delayed effects in stored product beetle adults have been also observed in the case of contact insecticides^34^. However, for some of the resistant populations tested here, the relationship between immobilization and phosphine concentration was not linear, showing a “peak-dip-peak” phenomenon, stimulating increased survival that, paradoxically, is exhibited between two peaks, which has been already described as a wider phenomenon^49,52^. In the case of contact insecticides, immobilization, which is more accurately referred as knockdown, may give insects the time that is required to detoxify the killing agent, through the termination of the exposure, leading to an increased recovery rather than increased delayed mortality levels^35,53,54^. Theoretically, this hypothesis may be true for phosphine, but has not been examined in detail so far^12,37^.

As in the case of delayed mortality, we have observed two peaks in the sweet spot in some of the combinations tested, suggesting different inhibition patterns, which may correspond to pesticide-mediated homeostatic modulation^55^. We also saw an interesting movement of the sweet spot both vertically, as a function of changes in exposures to the same concentration, and horizontally, which is mostly related with the population rather than the dose–response patterns. Unexpectedly, however, in the vast majority of the combinations tested here, the adults expressed their sweet spot in a similar way, regardless of the species, and the resistance status of the population. This spot is located at a rather stable exposure, that of 5 h, and a rather stable concentration, that of 1000 ppm, or in some cases, 2000 ppm. This stands in accordance with the initial data provided by Franco-Pereira et al.^48^ for the umbrella peak of a phosphine resistant *T. castaneum* population. The fact that many populations, both susceptible and resistant, exhibited similar sweet spots, may suggest that this phenomenon is correlated with the mode of action of phosphine and insects’ response, rather than a clearly hormetic pattern. Winks^36^ was the first to observe this non-linearity in *T. castaneum*, using the term “narcosis” after exposure to phosphine, rather than knockdown, which refers to movement deficiencies. In that study, the author clearly demonstrated that time to narcosis is determined by concentration, but only at certain concentration range, with a strong non-linear response. A series of follow-up tests by the same group for *T. castaneum*, displayed that this differential response to phosphine exposure is mostly manifested at short exposures, and at rather elevated concentrations, but is alleviated with a further concentration increase, showing a “protective stupefaction”^36,38^. In fact, Winks^36^ showed that this phenomenon was manifested in different populations of *T. castaneum*, and should not be considered as an outcome of previously existed resistance. This displays the existence of phosphine narcosis threshold that should be taken into account in dose–response bioassays, and differs sufficiently from immobilization after exposure^11,36,38^. Our data show that this threshold can be investigated more thoroughly at the concentration of 1000 ppm, when insects are exposed for relatively short intervals.

The exposure periods used here can be considered as rather short compared to “real world” applications of phosphine, as fumigation usually last for days, especially in the case of durable commodities. However, there are cases where phosphine is used for short intervals, as in the case of quarantine and pre-shipment treatments (QPS), in different export scenarios of fruits and vegetables^56,57^. Nevertheless, even in phosphine fumigations that are carried out in large structures, such as silos with grain bulks, the concentration of the gas changes dramatically over time, resulting in large areas to be partially treated, or even untreated^58–60^. In a recent study, Agrafioti et al.^60^ demonstrated that phosphine distribution within silos is rather uneven, and may leave large areas within the grain bulk that receives phosphine only for a short period of time, which may allow a part of the insect population to survive. Therefore, in commercial applications, there are cases which insects are exposed for short periods, which may trigger the aforementioned phenomenon. Although it is rare in small facilities and vessels, such as containers, this is also likely to occur, when fumigations are performed with high concentrations for short intervals. In commercial fumigations, Athanassiou et al.^61^ showed that phosphine concentrations of 1000 ppm or higher can be easily achieved, but if these concentrations remain for short periods (< 24 h), then insect survival is very likely to occur. The increased insect survival after short exposures to high phosphine concentrations may be related with resistance development, in the same way that resistance is linked with underdosing. Guedes et al.^47^ hypothesized that sublethal exposures may influence insecticide resistance beyond selection of resistant individuals, through insecticide-induced hormesis, but also via induction/cross-induction of detoxification enzymes. In this context, short exposures to elevated temperatures may trigger the “sweet spot” and lead to the development/selection of resistance, more rapidly than exposures to repeated applications of low/sublethal concentrations, which may delay selection for major single gene resistance^62^.

Our study demonstrates that both delayed mortality and the sweet spot are much wider phenomena than expected, as they apply to different stored product beetle species and populations. In populations of unknown resistance to phosphine, delayed mortality after short exposures can be further utilized as a means to estimate and further quantify resistance, as an addition to immediate responses after exposure to different diagnostic tests. Furthermore, the sweet spot triggers effects that are mostly due to a differential response of the insects, i.e. the narcosis threshold, which constitutes the crucial discrimination between narcotic and non-narcotic concentrations in dose–response bioassays. A further increase of the exposure time from up to 40 h tested here to longer intervals, e.g. 3 days is expected to alleviate extreme dissimilarities of insect control within a given population. On the other hand, short exposures to elevated concentrations, e.g. 1000 ppm or higher, is likely to increase these dissimilarities. Such a scenario is realistic and can occur in specific fumigation scenarios, such as in containers^63^. Failures in estimating the sweet spot thresholds may lead to false characterization of a population as resistant. Finally, short exposures to concentrations that are 1000 ppm or higher, apart from increased survival, may be related with rapid resistance development in stored product beetles, a hypothesis that merits additional investigation.

## Methods

### Insect cultures

We have used different populations of four species, with different susceptibility to phosphine (Table 7). From these populations, Lab *T. castaneum*, Lab *O. surinamensis* and Lab *R. dominica* were susceptible to phosphine (populations that have been maintained in the laboratory for more than 25 years with no exposure to phosphine) and 3TAB *S. oryzae*, BTS *T. castaneum*, ASC11 *O. surinamensis* and GA6 *R. dominica* were resistant to phosphine, based on a series of earlier bioassays^17,44^. All these populations were reared in the laboratory on wheat for *R. dominica* and *S. oryzae,* wheat flour for *T. castaneum,* and oat flakes for *O. surinamensis* (Table 7), at 26 °C, 55% relative humidity (RH) and continuous darkness. Only adults were used in the bioassays.

**Table 7:** Insect species and populations used in bioassays.

Population code	Species	Commodity reared	Origin
Lab	*Sitophilus oryzae*	Wheat	University of Thessaly
Lab	*Tribolium castaneum*	Wheat flour	University of Thessaly
Lab	*Oryzaephilus surinamensis*	Oat flakes	University of Thessaly
Lab	*Rhyzopertha dominica*	Wheat	University of Thessaly
3TAB	*Sitophilus oryzae*	Wheat	Germany
BTS	*Tribolium castaneum*	Wheat flour	Serbia
ASC11	*Oryzaephilus surinamensis*	Oat flakes	Greece
GA6	*Rhyzopertha dominica*	Wheat	Greece

### Evaluation of immobilization and recovery

In this bioassay, we used PTT, by exposing adults at different intervals, i.e. 15, 30, 60, 90, 150 and 300 min to 3000 ppm of phosphine, as suggested by Agrafioti et al.^17^. Briefly, ten adults of each of the tested populations were placed in a plastic syringe of 100 ml, with separate syringes for each population. The phosphine gas production took place inside a plastic canister of 5 l capacity, by adding two kit tablets and 50 ml of water. Concentration of the gas produced inside the canister was determined as suggested by Steuerwald et al.^31^ and a specific gas quantity was removed from the canister in order to reach the concentration of 3000 ppm into the syringe. After the termination of the exposure at the intervals mentioned above (separate bioassays for each interval), immobilization was measured and then the insects were removed from the syringe and transferred to petri-dishes with a small amount of food in each dish, i.e. cracked wheat (0.5 ± 0.1 g/dish) for *S. oryzae* and *R. dominica*, wheat flour (1.0 ± 0.1 g/dish) for *T. castaneum* and oat flakes (1.0 ± 0.1 g/dish) for *O. surinamensis*. The dishes were placed in an incubator set at 26 °C and 60% RH, and insect recovery, mortality and immobilization was recorded after 2 h, 1, 2 and 7 days. At these post-exposure periods, especially at 1, 2 and 7 d, we considered insect immobilization and not mortality, despite the fact that, depending on the population, the vast majority of the immobilized adults were dead. Two replicates with three sub-replicates were carried out for each combination (2 × 3 = 6 syringes per case).

### Occurrence of the “sweet spot”

We exposed adults of all species and populations at different exposures, i.e. 1, 3, 5, 20, 30 and 40 h and different concentrations, i.e. 500, 1000, 2000 and 3000 ppm. In brief, vials with ten adults of the tested populations were placed in an air-tight 1 l glass jar with separate vials for each combination of concentration -exposure interval. Using a glass syringe, the concentrations of phosphine mentioned above were taken from the freshly generated gas source (as described for the PTT protocol) and injected through a gas tight rubber septum of the 1 l glass jar with the test insects. After the termination of these intervals, the insects were placed in petri-dishes with food, and insect activity and immobilization/mortality were recorded 7 days later. In this series of bioassays, there were two replicates with two sub-replicates for each combination (2 × 2 = 4 jars per case).

### Data analysis

For the evaluation of immobilization and recovery, for each species, we compared the immobilization rates between the susceptible and resistant populations using the two-tailed *t*-test at the 0.05 level, for each exposure interval. The same procedure was followed for each of the post-exposure intervals. For each species, exposure time and post exposure time effects on immobilized adults were analyzed using a generalized linear model (GLM) which is more robust to violations of parametric assumptions, and run assuming a poison distribution and a logit link function, in Statistical Package for the Social Sciences (SPSS) Statistical Package (IBM SPSS v.25).

For the second series of bioassays, we used Probit Regression Analysis to estimate the lethal time, i.e. LT_99_ for each population and for each concentration. For species with different susceptibilities to phosphine, exposure time and concentration effects on immobilized adults were analyzed using a generalized linear model (GLM), assuming a poison distribution and a logit link function in SPSS. The same approach was followed for the delayed effect (after 7 days later). For both series of bioassays, curve fit estimation was provided by using the same statistical analysis, as stated above.
